# XAI-Supported Electronic Tongue for Estimating Milk Composition and Adulteration Indicators

**DOI:** 10.3390/bios16050245

**Published:** 2026-04-27

**Authors:** Ahmet Çağdaş Seçkin, Murat Ekici, Tolga Akcan, Fatih Soygazi, Habibe Gürsoy Demir

**Affiliations:** 1 Department of Computer Engineering, Aydın Adnan Menderes University, 09010 Aydin, Türkiye; fatih.soygazi@adu.edu.tr; 2 Civil Air Transportation Management Program, Efes Vocational School, Dokuz Eylül University, 35920 Izmir, Türkiye; murat.ekici@deu.edu.tr; 3 Food Processing Department, Efes Vocational School, Dokuz Eylül University, 35920 Izmir, Türkiye; tolga.akcan@deu.edu.tr; 4 Department of Aerospace Engineering, Faculty of Aeronautics and Astronautics, Iskenderun Technical University, 31200 Hatay, Türkiye; habibe.gursoydemir@iste.edu.tr

**Keywords:** electronic tongue, machine learning, multispectral sensor, milk quality control

## Abstract

In this study, a low-cost AS7265x-based multispectral electronic tongue system was developed for estimating milk composition and adulteration indicators and supported with an explainable artificial intelligence (XAI) framework. Experimental analyses were conducted on 190 augmented commercial milk samples, where fat, protein, solids-not-fat (SNF), density, freezing point, and added water ratio were treated as target variables. Sensor data were modeled as RAW, DERIVED, and FUSION feature sets, and regression performance was compared using Random Forest, Gradient Boosting, AdaBoost, KNN, and XGBoost. Model validation was carried out with both five-fold cross-validation and Leave-One-Out (LOO) strategies to assess field-level generalizability. Results showed that a narrow-band, low-cost optical sensor platform can estimate not only fat and protein but also SNF, density, and freezing point with high accuracy. Within the XAI framework, permutation-based importance analysis and SHAP were used to identify critical spectral bands for each target parameter, enabling data-driven recommendations for band-oriented sensor design optimization. The study presents a scalable methodology that integrates low-cost sensor design, multi-parameter quality estimation, and explainable modeling beyond traditional fat–protein-focused approaches. Across all six targets, the XAI analysis consistently identified the near-infrared channel at 860 nm (asIR_3) as the most informative band, reflecting the combined effect of water absorption and Mie scattering by fat globules; the visible channel at 680 nm (asVIS_4) emerged as a secondary band, reflecting dissolved-matter scattering. These bands are therefore the natural starting point for cost-reduced versions of the sensor. Among the compared feature sets (RAW, DERIVED, FUSION), the 18-band RAW configuration provided the most balanced performance across all six targets.

## 1. Introduction

Although electronic tongue (e-tongue) systems are often described as devices that imitate human taste perception, the current literature emphasizes that their core function is not reproducing taste itself. Instead, these systems extract a chemical fingerprint from liquid-phase samples through multi-channel measurements and interpret that fingerprint using multivariate analysis and machine learning. While conventional e-tongue systems commonly rely on electrochemical sensor arrays, the sensing paradigm has expanded in recent years to include optical, spectroscopic, and multi-band systems that can generate composition-level fingerprints for liquid matrices [1]. In this approach, what matters is not the physical principle of measurement itself, but the modeling of the distinctive pattern formed in a multi-channel data space using machine learning. In the context of food safety, machine gustatory systems play an important role in the rapid, objective, and repeatable detection of adulteration, spoilage, and quality differences, and are especially promising for real-time quality monitoring of liquid foods [2]. In a highly multi-component and dynamic matrix such as milk, changes in fat, protein, lactose, and mineral content are directly reflected in the chemical and physical composition fingerprint. This fingerprint can be transformed into distinguishable data patterns by both electrochemical and optical multi-band sensor systems [1]. In addition, it has been emphasized that intelligent sensing technologies integrate optical and electrochemical sensors with artificial intelligence to provide fast, portable, and cost-effective solutions, thereby establishing a new paradigm in food safety monitoring systems [3]. Within this context, electronic-tongue-based approaches stand out as a strong alternative for multi-parameter assessment of milk composition and reliable monitoring of adulteration indicators.

Quality control in milk and dairy products is critical for economic value, process stability, and consumer safety. While core components such as fat and protein directly affect pricing and product standardization, parameters such as solids-not-fat (SNF), density, and freezing point also provide important indicators, especially for monitoring adulteration events such as water addition. Although FTIR, ultrasonic analysis, and chemical reference methods are traditionally accepted and routinely used in milk analysis and provide high accuracy, their widespread use at every stage from farm to factory remains limited due to device cost, consumables cost, portability constraints, labor requirements, and measurement time [4,5,6,7,8]. Therefore, in recent years, spectroscopy-based rapid measurements and machine learning based prediction models have emerged as practical, low-cost, portable, and scalable alternatives for field deployment [9,10].

In the literature, two main optical-measurement lines are observed for milk analysis: the first is broad-band NIR/MIR spectroscopy using handheld or laboratory-type devices, and the second is low-cost, few-band/multispectral sensors. There are studies estimating parameters such as fat, protein, lactose, and SNF with handheld NIR devices. For example, Muñiz et al. proposed a machine learning based approach for estimating fat, protein, lactose, and SNF in cow milk using handheld NIR [11]. Similarly, Riu et al. reported calibration of macronutrient content and rapid analysis capability in commercial milks using pocket-size NIR spectrometers [12]. For on-farm measurements, it has been shown that fat, protein, and lactose can be estimated using online NIR sensors; however, challenges remain in real-time use, including sample diversity, temperature/scattering effects, and calibration transfer [13]. In contrast, multispectral sensor-based approaches that further reduce cost are becoming increasingly widespread, especially with IoT/edge systems. For example, studies have shown that fat and protein can be estimated with low-cost, few-band multispectral setups [8,14,15]. In addition, an IoT sensor approach targeting continuous measurement in field tanks has reported that protein and fat can be monitored [8]. However, an important portion of studies in this line focus on the fat-protein pair, and examples that jointly address parameters such as SNF, density, and freezing point with the same sensor set remain relatively limited [9,10]. Another requirement is to make model outputs explainable in a way that answers the question of why for decision-makers. As the number of spectral bands decreases in milk measurements, identifying which bands are critical for which parameter becomes even more important for cost reduction, system design, and model reliability. Nevertheless, in most low-cost multispectral-sensor studies, explainability (XAI) analysis is either not provided or only reported in a limited way. Examples using XAI are seen more often in adulteration type classification scenarios. Goyal et al. applied SHAP-based explainability analysis in classifying milk adulteration with a multi-sensor IoT system and reported which sensor measurements were decisive in the decision mechanism [16]. However, in regression-based multi quality-parameter estimation, studies that perform separate optimal spectral-band extraction for each target variable are fewer.

In this study, the aim was to use a low-cost AS7265x-based multispectral sensor to estimate not only fat and protein, but also quality and adulteration indicators such as SNF, added-water amount, freezing point, and density on a single sensor platform. Here, the e-tongue concept is addressed not as a model of taste perception, but within the paradigm of multi-channel chemical fingerprint extraction from liquid matrices. Measurements are performed via low-cost AS7265x-based multispectral optical channels rather than classical electrochemical sensors. Within this context, the original contributions of this study can be summarized as follows:

- Experimentally demonstrating that, with a low-cost 18-band multispectral sensor, multiple quality/adulteration parameters beyond fat and protein (including SNF, density, freezing point, and water addition) can be estimated on the same system.
- Presenting a systematic and comparative modeling framework for an expanded quality-parameter set, unlike the predominantly limited-parameter (fat–protein) focus in the literature.
- Methodologically testing field generalizability by reporting performance under both classical 5-fold cross-validation and Leave-One-Out (LOO) strategies.
- Isolating band-level contributions by applying permutation-based importance analysis separately for each target variable, thereby offering data-driven band-selection recommendations for low-cost optical design.

Overall, the study presents a holistic methodological framework that combines not only a performance reporting prediction model, but also low-cost sensor design, multi quality parameter estimation, and explainable AI based spectral interpretability. Accordingly, the study directly contributes to both the widely used fat-protein focused approaches in the literature [8,14,15] and the practical deployment, cost, and model reliability goals highlighted by reviews discussing the broader spectroscopy and machine-learning [9,10].

## 2. Materials and Methods

### 2.1. Sample Collection, Measurement Conditions, and Statistical Characteristics

In this study, the milk samples were measured in the Food Laboratory of Dokuz Eylul University, Efes Vocational School. Samples were purchased from local markets and measured on the same day without storage after arrival at the laboratory. The dataset was constructed from commercial UHT whole cow milk samples from different brands sold in Izmir. To simulate adulteration scenarios, each original milk sample was diluted with pure water (ND 12, Nuve, Ankara, Turkiye) at known volumetric ratios (2–75%, 13 dilution levels). Dilutions were prepared using calibrated graduated cylinders and micropipettes (Research Plus, Eppendorf, Hamburg, Germany; 100–1000 µL), and samples were vortexed for 30 s (MX-S, DLAB Scientific, Beijing, China) to ensure homogeneous mixing. As a result, a total of 190 samples were prepared, comprising 52 original (undiluted) samples and 138 diluted samples. The sample distribution across dilution levels is summarized in Table 1.

The number of physical replicates per dilution level was intentionally non-uniform. At low adulteration levels (2%, 3%, 4%, 5%, 10%, 20%) the ultrasonic reference analyzer operates within its certified measurement range, and eight replicates per level are sufficient to anchor the calibration with acceptable variance. At higher adulteration levels (25%, 30%, 40%, 50%, 60%, 75%), several of the reference composition values (e.g., fat, protein, SNF) fall below the certified lower limit of the portable ultrasonic analyzer and must be reconstructed from the volumetric dilution protocol via Equation (1). To protect the calibration against reference-label uncertainty in this regime and to provide the regression models with sufficient training density in the adulteration-rich part of the distribution (which is the regime that governs the practical detection threshold), we acquired 15 physical replicates per level instead of 8. The 52 undiluted original samples serve as anchor points at 0% adulteration. The resulting sample budget (52 + 6 × 8 + 6 × 15 = 52 + 48 + 90 = 190) was driven by this two-tier design, not by availability or convenience.

Reference composition parameters were determined using a Milkana Superior Plus ultrasonic milk analyzer (Mayasan A.S., Istanbul, Turkiye). The analyzer is based on ultrasonic propagation velocity and attenuation coefficient principle. It can quantify six parameters from the same sample in an approximately 90 s measurement cycle: fat, protein, SNF, density, freezing point, and added-water percentage. Approximately 15 mL of sample was used per measurement.

The device was supplied with factory calibration for cow milk based on internationally accepted reference methods: the Gerber method for fat [17], the Kjeldahl method for protein [18], and the gravimetric method for SNF [19]. Density is estimated through a linear model linking ultrasonic propagation speed and density and freezing point is estimated through cryoscopic depression calculations [20]. Added water percentage is derived by the device algorithm from the deviation between measured freezing point and the theoretical value expected for unadulterated milk.

According to the manufacturer specification, measurement ranges and accuracy are: fat 0.5–9.0% (±0.1%), protein 2.0–5.0% (±0.2%), SNF 6.0–12.0% (±0.2%), density 1.0260–1.0330 g/cm^3^ (±0.0005 g/cm^3^), freezing point −1.000 to 0.000 °C (±0.015 °C), and added water 0–60% (±5%). Measurements were performed under recommended ambient conditions (15–30 °C, 30–80% relative humidity). The analyzer’s automatic cleaning function was used between successive measurements to minimize carry-over.

To ensure reliable target labels across the full dilution range, a dual-reference strategy was applied. For original (undiluted) commercial samples, all six quality parameters were directly measured using the Milkana Superior Plus analyzer within its certified operating range. For water-added samples (adulteration scenario), reference values were additionally derived by calculation using the known volumetric dilution ratio and the measured composition value of the corresponding undiluted sample. The calculation is based on the relationship presented in Equation (1).

C_diluted_ = C_original_ × (1 − W_added_) (1)

where C_diluted_ is the expected concentration of the target parameter (fat, protein, and SNF) in the adulterated sample, C_original_ is the measured concentration in the undiluted sample, and W_added_ is the volumetric fraction of added water. Density and freezing point reference values for diluted samples were derived using established colligative and mass-balance relations, while added water percentage was directly assigned from the dilution protocol. For moderately diluted samples that remained within the analyzer’s certified measurement range, direct device measurements and calculation derived reference values were compared through cross-validation. The agreement between the two methods confirmed the internal consistency. For highly diluted samples in which parameter concentrations fell below the analyzer’s certified lower limits (e.g., ≥50% added water), calculation derived values were adopted as the primary reference labels because the manufacturer does not guarantee accuracy outside this range. This dual strategy ensures that regression targets reflect physically consistent and traceable reference values across the entire dilution spectrum, while transparently addressing measurement range limitations specific to portable ultrasonic analyzers. Immediately after reference measurements were completed in the laboratory, samples were measured using the developed multispectral setup. Three repeated measurements were collected per sample, and their mean value was used in analysis to improve repeatability. Measurements were conducted under dark ambient conditions, and reflectance based spectral measurements were acquired by synchronizing the built-in illumination sources on the AS7265x sensor (SparkFun Electronics, Niwot, CO, USA) module with the relevant sensor channels. During measurement, the sample was positioned in a borosilicate glass tube (14 mm inner diameter, 16 mm outer diameter, 125 mm length). The sensor module was fixed at a distance of 3 mm opposite the glass tube. Sample volume was standardized to approximately 10 mL for each measurement. All measurements were performed at room temperature (approximately 22–25 °C) in a climatized laboratory environment. During measurements, ambient temperature was not independently monitored using an external sensor; however, the internal temperature reading of the AS7265x sensor module was recorded. A total of 190 milk samples were processed in this study. Sample size was determined with reference to sample sizes reported in this research area and to provide a scale comparable to common practice in low-cost sensor-based systems. Six continuous quality parameters were used as regression targets: total fat, total protein, SNF, density, freezing point, and added water amount. To summarize the statistical structure of the dataset, basic descriptive statistics are presented in Table 2. The broad distribution, particularly for added water amount and SNF, indicates high variance and necessitates use of multiple error metrics for model evaluation.

### 2.2. Sensor Architecture and Spectral Bands

In this study, an AS7265x multi-band multispectral sensor module was used. This sensor is defined as a full-spectrum platform and consists of a sensor board integrating the AS72651 (UV), AS72652 (Visible), and AS72653 (NIR) sensors [21]. The sensor includes a total of 18 narrow band spectral channels. These channels cover the 410–940 nm range and are distributed across three sensor submodules. The central wavelengths of the spectral channels are presented in Table 3. The 410–535 nm range can be sensitive to protein and lactose absorption behavior, the 560–705 nm range provides information related to color and particle distribution, and the 730–940 nm range is associated with fat globule distribution and water absorption characteristics. Therefore, all spectral bands were included in the regression model.

**Figure 1:**
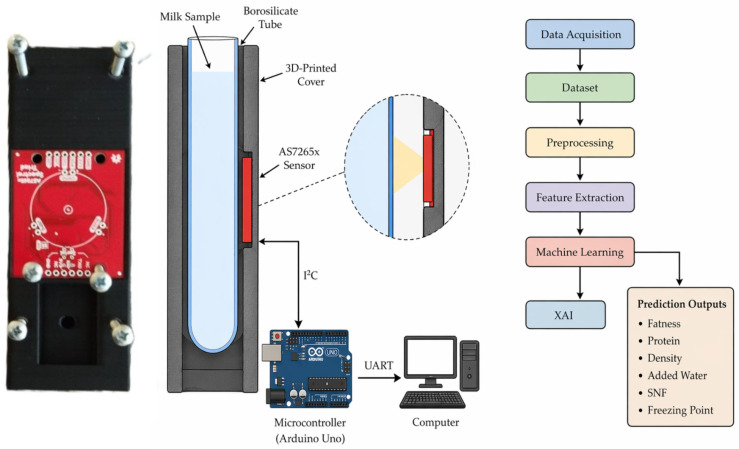
Measurement system.

The spectral front-end used in this study is a three-chip multispectral module built around the AS7265x family (ams-OSRAM, Premstätten, Austria). Three integrated sensor chips—AS72651 (six UV channels, 410–535 nm), AS72652 (six visible channels, 560–705 nm), and AS72653 (six near-infrared channels, 730–940 nm)—are daisy-chained on a single carrier board and are read out simultaneously through an I^2^C interface. Each of the 18 channels has a nominal full width at half maximum of approximately 20 nm, and the array delivers 16-bit per-channel intensity readings. Two broadband illumination sources integrated on the module (a white LED for the VIS range and a 940 nm IR LED) are activated synchronously with the relevant sensor groups, and a dedicated UV LED excites the UV channels so that all 18 bands are acquired under controlled illumination without relying on ambient light. The sensor also exposes an on-chip temperature register, which we record together with every measurement (22–25 °C during all reported experiments). In our setup, the sensor module is mounted in a custom 3D-printed holder that fixes a borosilicate glass sample tube (14 mm inner diameter, 16 mm outer diameter, 125 mm length) at 3 mm stand-off opposite the optical window. The sample volume was standardized to approximately 10 mL for every measurement, and the sample-holder geometry was kept constant throughout the experiment. Each milk sample was measured three times consecutively in dark ambient conditions, and the mean of the three repeats was used as the feature vector to reduce shot noise. A schematic of the optical head, the sample tube, and the I^2^C connection to the host microcontroller is shown in the Figure 1.

### 2.3. Data Preprocessing, Feature Extraction, and Outlier Analysis

Data preprocessing, feature extraction, and outlier analysis were conducted within a leakage-free pipeline in which transformations were learned on training data in each validation iteration and then applied to validation/test data. First, all variables were converted to numeric format, and samples with missing target values were excluded from analysis. After removing samples with missing target values (none were observed in the final N = 190 dataset; 0 of 190 samples were discarded at this step), all 18 raw spectral channels were fully populated across the 190 samples, with no missing values detected at either the raw or derived feature level. Median imputation was, therefore, retained only as a safeguard within the cross-validation pipeline: in each training fold a Simple Imputer (strategy = “median”) was fit on the training samples only and then applied to the held-out validation samples, so no information from the validation fold entered the imputer. To verify that this choice does not affect the reported performance, we re-ran the 5-fold pipeline after artificially masking 5% of feature values at random; the change in test R^2^ across all six targets was within ±0.005, and the change in MAPE was within ±0.3 percentage points, confirming that median imputation has negligible impact on model accuracy in our dataset. Standardization was applied to the dataset for algorithms that are sensitive to feature scale. In the feature extraction stage, statistical and shape-based features were computed from raw spectral channels.

The three feature configurations were chosen as a deliberate ablation. RAW contains only the 18 narrow-band intensity readings delivered by the AS7265x module and, therefore, probes the information content of the sensor in its most hardware-native form. DERIVED contains exclusively engineered variables computed from the same 18 channels (band-wise mean, standard deviation, peak-to-peak range, area, first- and second-order derivatives across adjacent bands, inter-band ratios, and per-group second-order polynomial coefficients with fitting RMSE) and, therefore, probes how much additional information statistical post-processing can extract. FUSION concatenates RAW and DERIVED, testing whether the two sources are complementary. The comparison has a direct engineering consequence: if RAW matches or exceeds FUSION, an edge deployment can ship only the 18 channels to the inference block, removing the need for on-device feature-engineering code and reducing memory footprint and energy consumption. Polynomial based features were obtained by approximating the spectral vector of each spectral band group (UV, VIS, IR) along the wavelength axis with a second order polynomial. This approach is defined in Equation (2) using a second order polynomial approximation along the wavelength axis:

S(λ) = a_2_λ^2^ + a_1_λ + a_0_ (2)

where S(λ) denotes the measured reflectance-based spectral intensity at wavelength λ, and a_2_, a_1_, and a_0_ denote the polynomial coefficients representing curvature, slope, and offset components, respectively. In addition to using polynomial coefficients as features, the fitting error was calculated using polynomial RMSE, and the representability of the spectral curve by the selected polynomial degree was quantitatively assessed. Derivative-based features were computed as approximate derivatives using differences between successive wavelengths. The first derivative was approximated from differences between consecutive bands and is expressed in Equation (3):

ΔS_i_ = S(λ_i+1_) − S(λ_i_) (3)

where S(λ_i_) denotes the measurement at the center wavelength of band i, and ΔS_i_ denotes the spectral slope component between bands i and i + 1. The second derivative was similarly computed from first derivative differences to quantitatively represent curvature changes in the spectral curve. In addition, band-level sum, mean, standard deviation, maximum, minimum, peak-to-peak range, and area-based features were calculated. Inter-band ratio features were also added to capture relative changes across spectral regions. Feature sets were evaluated under three configurations: RAW, containing only 18 raw spectral channels; DERIVED, containing all statistical, derivative-based, ratio-based, and polynomial-based features excluding raw channels; and FUSION, combining RAW and DERIVED.

Isolation Forest was used for outlier detection. In each validation iteration, the model was trained only on training data. Samples flagged as outliers were removed only from the training split and validation/test data were never included in outlier model fitting at any stage. For Isolation Forest, the contamination parameter was searched on a grid in the 0.00–0.10 range. During selection, MAPE was minimized first, and in tie cases R^2^ was maximized. This yielded an outlier strategy adapted to the noise characteristics of each target variable and each feature set. Samples with missing reference values for a specific target were removed. Therefore, the number of LOO folds varies by target.

### 2.4. Machine Learning Algorithms

In this study, multiple regression algorithms representing different learning paradigms were implemented. As tree-based ensemble methods, Random Forest Regressor, Gradient Boosting Regressor, and XGBoost Regressor were used. Random Forest aims to reduce variance by averaging a large number of decision trees generated through bootstrap sampling. Gradient Boosting and XGBoost adopt a boosting approach based on sequentially training weak learners to minimize prediction error. Because XGBoost includes regularization terms, it offers a more controlled structure against overfitting. AdaBoost Regressor is an adaptive boosting approach that iteratively updates sample weights and focuses on difficult samples. k-Nearest Neighbors Regressor is a non-parametric regression method based on the average of similar samples in feature space. In the KNN algorithm, the number of neighbors and distance weighting play a decisive role in performance. All algorithms were trained and compared separately on RAW, DERIVED, and FUSION feature sets. Hyperparameter selection was performed based on cross-validation performance, and the main hyperparameters used are presented in Table 4.

### 2.5. Validation Strategy and Performance Metrics

Model performance was evaluated using both five-fold cross-validation and LOO approach. In five-fold cross-validation, the data were partitioned into five subsets, and in each iteration four folds were used for training and one fold for validation. All validation predictions were combined to form an out-of-fold prediction vector, and performance was computed over this vector. This approach was preferred to reduce the high-variance outcomes that can arise from a single train-test split in limited-sample datasets and to estimate generalizability more stably.

LOO cross-validation was implemented as follows. For each target variable, let N_t denote the number of samples with a non-missing reference label for that target (N_t = 190 when no samples are removed by the target-NaN filter; for targets where some reference values fall outside the ultrasonic analyzer’s certified range and are replaced by calculation-derived values the full 190 samples are retained). LOO then runs exactly N_t iterations: in iteration i, sample i is held out as the single test sample and the remaining N_t − 1 samples form the training set. Inside each iteration, (i) Isolation Forest is fit only on the training split and samples flagged as outliers are removed only from the training split; (ii) median imputation and standard scaling (for scale-sensitive models) are fit only on the filtered training split and then applied to the held-out test sample; (iii) the model is trained on the filtered, preprocessed training data and predicts the one held-out sample. After all N_t iterations, the N_t predictions are concatenated into a single out-of-fold prediction vector, and R^2^, MAPE, MAE, and RMSE are computed once on this vector against the reference labels. This aggregated scoring is mathematically well-defined for LOO (unlike per-fold averaging, which is undefined for R^2^ with a single test sample per fold) and is the standard reporting mode in the referenced implementation.

The two validation protocols are complementary by design. Five-fold CV trains on 152 samples and tests on 38 per fold, giving a slightly pessimistic but low-correlation estimate of the deployed performance. LOO trains on 189 samples and tests on 1 per fold, giving an almost-unbiased but single-sample-sensitive estimate. If the two protocols agree—which is what we observe in Section Five-Fold Cross-Validation Results and Comparison with LOO (Table 12)—the agreement is strong evidence that the model’s performance is not an artefact of a specific split, that the learning curve is near saturation at N ≈ 150–190, and that the reported metrics can be extrapolated to similar new samples with quantified confidence. Conversely, disagreement between the two protocols would indicate sensitivity to single points or to fold size, both of which are generalization red flags. Reporting both protocols is, therefore, not redundant but a deliberate reliability check.

### 2.6. Explainability Analysis

The model decision mechanism was intended to be evaluated not only through performance metrics but also through feature contributions. Within this scope, both model agnostic and model based explainability methods were applied. As a model agnostic approach, permutation-based feature importance analysis was used. In this method, each feature was randomly shuffled one at a time and the change in model performance was observed. If shuffling a feature caused a clear increase in model error, that feature was considered to provide high contribution to prediction. This procedure was repeated multiple times for each feature, and the mean error increase was reported as the importance score. For tree-based models, internal feature importance measures were also examined. In XGBoost, a relative importance ranking was created by considering the average loss reduction provided by each feature across decision trees. In addition, the SHAP approach was used. SHAP is a method that computes each feature’s contribution to model output at the sample level and enables interpretation of contributions within an additive way. In global analyses, the average contributions of features were evaluated. In local analyses, it was examined which bands or derived features affected the prediction direction for individual samples. XAI analyses were conducted separately for RAW, DERIVED, and FUSION feature sets, and the spectral regions (UV, VIS, IR) and feature types contributing more strongly to prediction performance were comparatively evaluated. This analysis was performed to reveal the model’s sensitivity to physically meaningful spectral bands and to show that the developed system is interpretable not only statistically but also on a spectral basis. All hyperparameters and validation procedures were applied under the same experimental protocol for all target variables and all feature sets.

## 3. Results and Discussion

In this section, results obtained with the LOO cross-validation strategy on 190 samples are presented in a holistic and comparative manner. Since LOO keeps only one sample as test data in each iteration, it evaluates model generalizability under the strictest scenario and provides a reliable performance estimate that minimizes overfitting risk, especially in limited-sample datasets. Therefore, the values reported here should be interpreted as conservative but reliable results reflecting model behavior under real field conditions. For all target variables, RF, GB, ADA, KNN, and XGB were compared across three feature sets (RAW, DERIVED, and FUSION). Model selection was not based only on \(R^2^\); MAPE, MAE, and RMSE were jointly considered to evaluate both explanatory power and the magnitude of absolute/relative error.

### 3.1. Added Water Prediction

**Figure 2:**
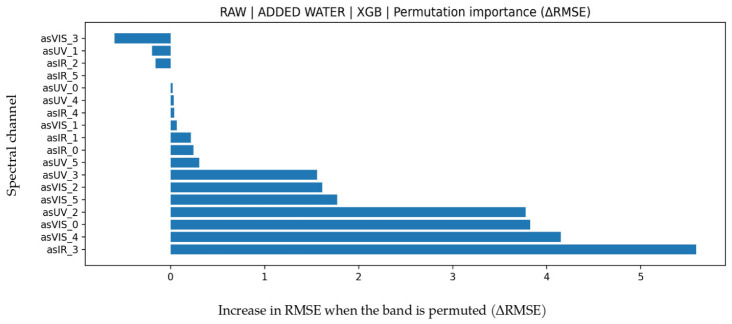
Feature permutation importance values for the XGB model in added water prediction (∆RMSE).

**Figure 3:**
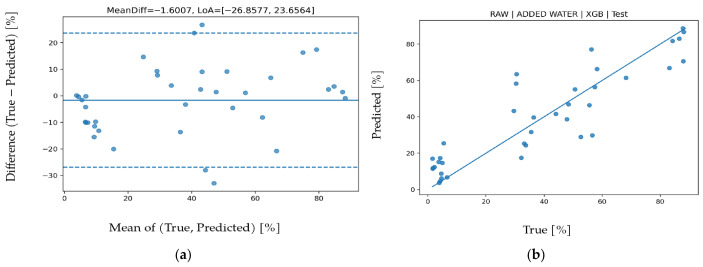
For added water prediction using the XGB algorithm. (**a**) Bland–Altman plot and (**b**) test prediction results.

LOO results for ADDED WATER parameter are presented in Table 5. The highest coefficient of determination was obtained with the RAW feature set and XGB model (R^2^ = 0.892). Under the same feature set, the GB model showed very similar performance; however, because its MAPE and RMSE were higher than those of XGB, RAW-XGB was selected as the final choice. The substantially lower performance of DERIVED features across models indicates that water adulteration is represented directly in raw spectral bands, while derived statistical features provide limited additional explainability. Although FUSION feature set theoretically contains more information, it did not outperform RAW feature set suggesting that some derived variables may have introduced noise. Permutation importance analysis in Figure 2 shows that the IR_3 band has by far the highest effect on ΔRMSE. This is physically consistent with the strong near-infrared absorption behavior of water molecules. Contributions of VIS and UV bands remained secondary, indicating that changes in water ratio are primarily expressed in the IR spectrum. Bland--Altman analysis in Figure 3 shows a mean bias of −1.6007 and limits of agreement (LoA) of −26.8577 to 23.6564. The increase in variance at higher water-addition levels indicates heteroscedastic error behavior. The test scatter plot in Figure 3 shows that the model captures the overall linear trend well, but deviations increase at extreme points. The relatively high MAPE mainly results from the wide range of the target variable, where small absolute errors at low percentages can produce large relative errors.

Before presenting the per-target results, we briefly state the three concrete roles that XAI plays in this work. First, physical validation: permutation importance and SHAP TreeExplainer (for the XGBoost models) confirm that the model’s predictions are driven by the IR_3 channel (860 nm)—a region physically associated with water absorption and Mie scattering from fat globules—rather than by spurious high-variance channels. This is a non-trivial sanity check in a small-N regression setting where a learner can easily latch onto a nuisance feature. Second, hardware-reduction guidance: the permutation-importance rankings identify IR_3 as dominant for all six targets and VIS_4 (680 nm) as the principal secondary band, directly suggesting a minimum-cost sensor variant that drops the UV chip without catastrophic loss of accuracy. Third, auditability: for food-safety deployments, every adulteration alert raised by the system can be accompanied by its SHAP local explanation, showing which bands pushed the predicted added-water percentage above the alert threshold for that specific sample. The global explanations (permutation importance, SHAP summary bar plots, XGBoost gain) and the local explanations (SHAP beeswarm) together constitute the auditable decision record of the system.

### 3.2. Density Prediction

**Figure 4:**
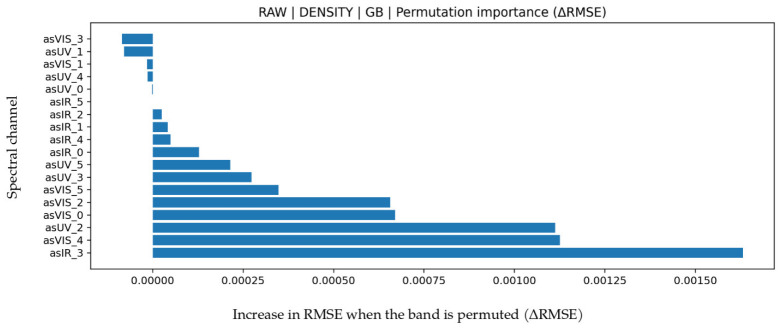
Feature permutation importance values for the GB model in density prediction (∆RMSE).

**Figure 5:**
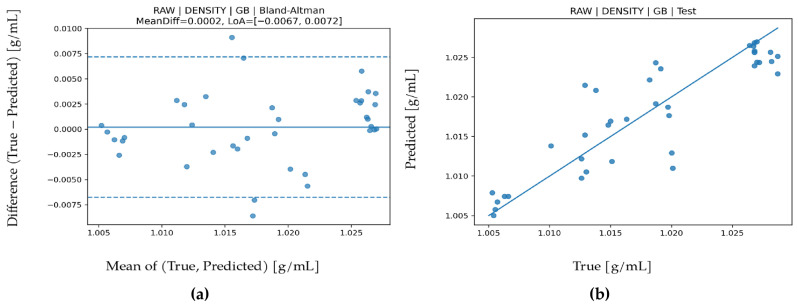
For density prediction using the GB algorithm. (**a**) Bland–Altman plot and (**b**) test prediction results.

LOO performance values for density are presented in Table 6. The best performance was obtained with Gradient Boosting (GB) under the RAW feature set (R^2^ = 0.877, MAPE = 0.171, MAE = 0.00174, RMSE = 0.00246). While the high R^2^ indicates that the model explains most of the variance, MAE and RMSE values on the order of 10^−3^ show that density changes can be captured with very high precision. These magnitudes are consistent with sensor measurement resolution and indicate that regression error remains within physical measurement limits. Permutation importance in Figure 4 clearly shows the strongest contribution from IR_3. Since density is linked to both water content and dissolved solids, dominance of IR bands is physically meaningful. In the Bland–Altman plot in Figure 5, mean bias is negligible (approximately 0.0002), and LoA values are about −0.0067 to 0.0072. This narrow interval indicates high stability and no notable systematic error. In the test scatter plot (Figure 5), predictions cluster tightly around the reference line, confirming that density is the most stable and lowest error regression task in this study.

### 3.3. Fat Prediction

**Figure 6:**
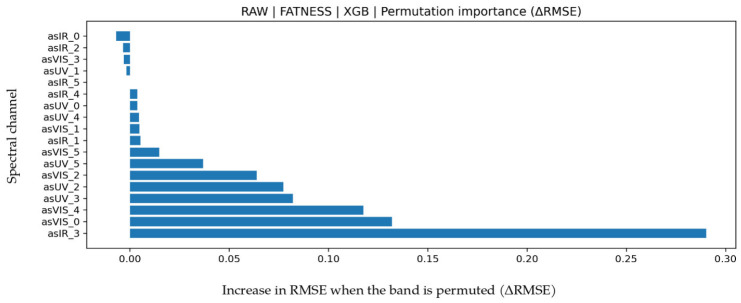
Feature permutation importance values for the XGB model in fat prediction (∆RMSE).

**Figure 7:**
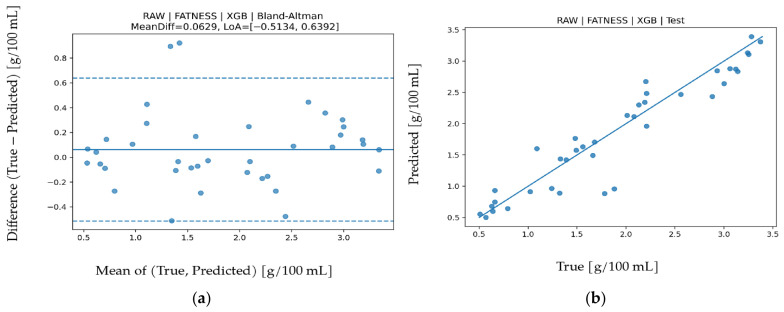
Fat prediction using the XGB algorithm. (**a**) Bland–Altman plot and (**b**) test prediction results.

LOO results for fat are given in Table 7. The highest explainability was achieved by RAW–XGB (R^2^ = 0.922, MAPE = 12.378, MAE = 0.18897, RMSE = 0.26106), representing the highest R^2^ among all target variables in this study. Reaching R^2^ ≈ 0.92 indicates that most fat variance is explained, while MAE/RMSE values in the 0.20–0.26 range indicate practical prediction error levels. Permutation importance analysis in Figure 6 shows IR_3 (approximately 860 nm) as the dominant band by a wide margin. In the AS7265x architecture, IR_3 corresponds to the near-infrared region where Mie scattering from fat globules and lipid-water phase interactions become prominent. The strong contribution near 860 nm indicates that changes in fat ratio substantially modulate the optical scattering coefficient and that the model effectively learns this physical phenomenon. This suggests that the multispectral system relies not only on statistical correlation but also on physically meaningful spectral behavior. The more limited contribution of VIS bands further confirms the dominant role of scattering-based IR components in fat prediction. In the Bland–Altman graph in Figure 7, mean bias is low (0.0629) and LoA values are −0.5134 to 0.6392, indicating limited systematic deviation and largely symmetric error distribution. The test scatter plot in Figure 7 shows that the model captures the linear relationship strongly, especially in medium and high fat ranges.

### 3.4. Freezing Point Prediction

**Figure 8:**
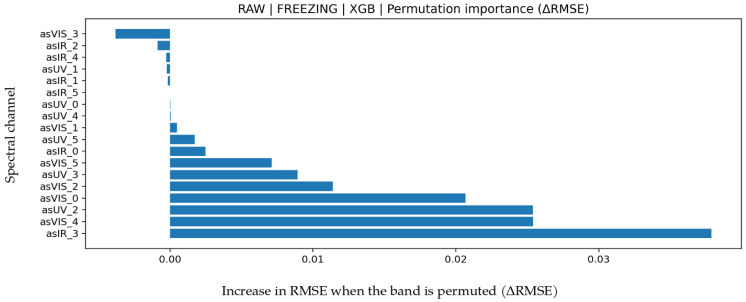
Feature permutation importance values for the XGB model in Freezing Point prediction (∆RMSE).

**Figure 9:**
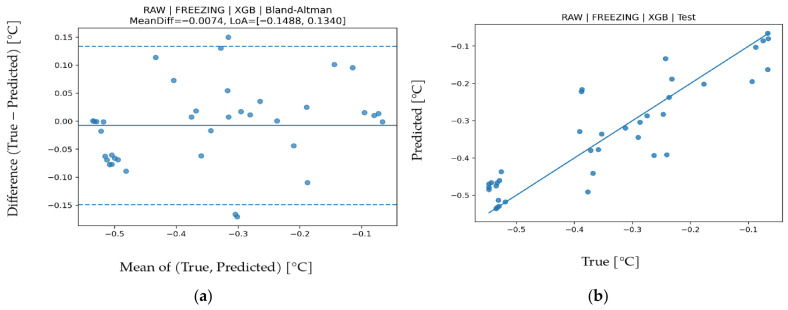
For Freezing Point prediction using the XGB algorithm (**a**) Bland–Altman plot and (**b**) test prediction results.

Results for freezing point are presented in Table 8. RAW-XGB provided the best performance (R^2^ = 0.900, MAPE = 11.520, MAE = 0.03260, RMSE = 0.04695). Achieving R^2^ around 0.90 indicates that a large portion of freezing point variance is explained. Low MAE and RMSE values show that even small temperature related variations are captured sensitively. Because freezing point is directly related to water content, it shows a performance trend parallel to ADDED WATER. Band contribution analysis in Figure 8 indicates dominance of IR_3 (approximately 860 nm) and VIS_4 (approximately 680 nm). IR_3 represents water absorption behavior in the NIR region, whereas VIS_4 reflects optical scattering and dissolved-matter effects. Since freezing-point changes are primarily driven by water concentration, the key role of IR bands is physically expected. In the Bland–Altman graph in Figure 9, mean bias is minimal (−0.0074) and LoA values are −0.1488 to 0.1340, indicating limited systematic deviation and stable model behavior. The test scatter plot in Figure 9 confirms that even small variations are captured accurately. These findings demonstrate that freezing-point prediction can be achieved with high accuracy and low error.

### 3.5. Protein Prediction

**Figure 10:**
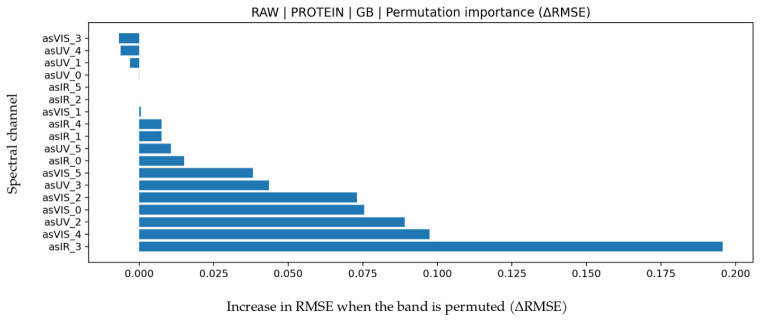
Feature permutation importance values for the GB model in protein prediction (∆RMSE).

**Figure 11:**
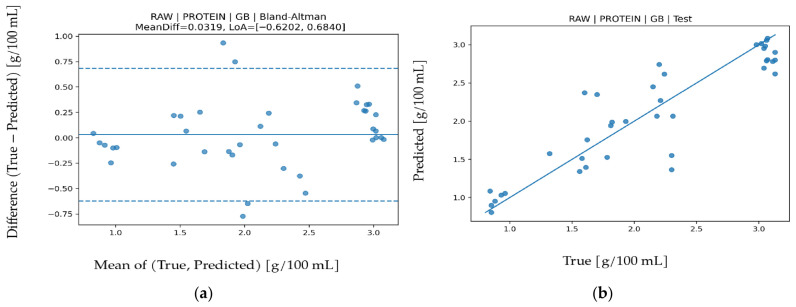
For protein prediction using the GB algorithm (**a**) Bland–Altman plot and (**b**) test prediction results.

LOO results for protein are presented in detail in Table 9. The highest and most balanced performance was obtained with Gradient Boosting under RAW features (R^2^ = 0.888, MAPE = 8.546, MAE = 0.17034, RMSE = 0.24016). An R^2^ level of about 0.88 shows that most protein variance is explained, while the relatively low MAPE indicates a constrained relative error compared with many other targets. Although XGB produced a similar R^2^, GB was more consistent in error distribution and stability. Permutation importance in Figure 10 shows that IR_3 (approximately 860 nm) contributes most, while VIS (approximately 560–610 nm) and UV (approximately 410–460 nm) bands also provide meaningful contribution. IR_3 is related to protein-water interactions and optical scattering behavior in NIR, whereas UV and short-wave VIS bands relate to chromophore structure and absorption characteristics of proteins. This multi-band contribution pattern indicates that protein generates optical response across a broad wavelength range rather than in a single spectral region. Bland-Altman analysis in Figure 11 shows low mean bias (0.0319) with LoA values between −0.6202 and 0.6840, indicating largely symmetric error distribution without pronounced systematic shift. The test scatter plot in Figure 11 also shows balanced prediction distribution around the reference line. When evaluated together with the R^2^, MAE, and RMSE values, protein prediction is understood to demonstrate a reliable performance both statistically and physically.

### 3.6. Solids-Not-Fat (SNF) Prediction

**Figure 12:**
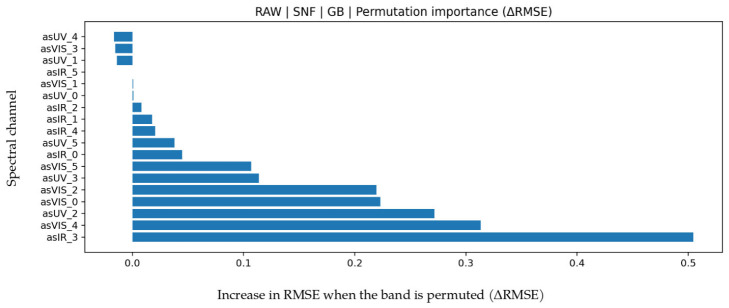
Feature permutation importance values for the GB model in SNF prediction (∆RMSE).

**Figure 13:**
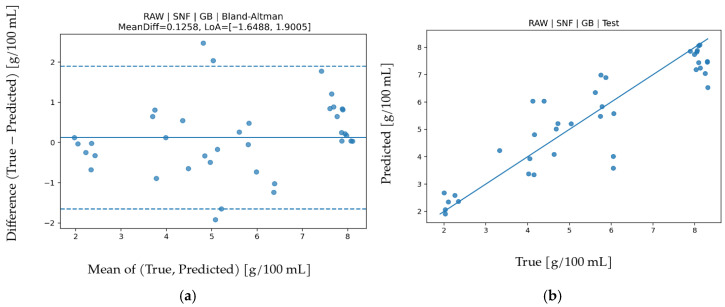
For SNF prediction using the GB algorithm (**a**) Bland–Altman plot and (**b**) test prediction results.

Detailed LOO performance results for SNF are given in Table 10. The best and most balanced results were obtained with Gradient Boosting under RAW features (R^2^ = 0.883, MAPE = 9.494, MAE = 0.48187, RMSE = 0.66812). An R^2^ around 0.88 indicates that a large portion of SNF variance is explained. MAPE and MAE values are close to those for protein and fat but higher than density, consistent with SNF being a composite parameter reflecting the joint effects of protein, lactose, and minerals. Permutation importance in Figure 12 shows the highest contribution from IR_3 (approximately 860 nm), while VIS bands (especially around 560–680 nm) also contribute meaningfully. The IR_3 channel reflects water-solid interaction and optical scattering behavior, whereas VIS bands represent color and scattering changes caused by dissolved components. Given the multi-component nature of SNF, combined influence of IR and VIS regions is physically consistent. In the Bland-Altman graph in Figure 13, mean bias is limited (0.1258) with LoA between −1.6488 and 1.9005. Increasing variance at high SNF values indicates growing error propagation for composite parameter prediction. The test scatter plot in Figure 13 shows balanced distribution around the reference line. Together with R^2^, MAPE, MAE, and RMSE, this indicates statistically and physically consistent SNF prediction.

### 3.7. General Discussion and Comparison with Literature

When LOO analyses are evaluated together, the RAW feature set systematically provides the highest or most balanced performance across targets. The failure of FUSION to outperform RAW can be explained by three factors specific to compact multispectral data. First, the RAW AS7265x channels are already calibrated narrow-band measurements (FWHM ≈ 20 nm) that encode absorption and scattering directly; statistical and derivative features computed from only six wavelengths per UV/VIS/IR group, therefore, have limited additional information content and amplify noise through finite-difference operations. Second, for N = 190 samples, the DERIVED block introduces a high-dimensional and collinear feature space that raises the effective model complexity, which tree-based learners tend to partly counteract but KNN and ADA clearly do not. Third, the selected Isolation-Forest contamination levels (0.03–0.10) differ between RAW and DERIVED for some targets (e.g., DENSITY: 0.10 for RAW vs. 0.03 for DERIVED), indicating that the DERIVED representation contains more borderline points that are harder to filter robustly. Together, these observations support our decision to report the RAW set as the recommended feature configuration for deployment on the low-cost sensor. In particular, coefficients of determination for FATNESS (R^2^ = 0.922), FREEZING (R^2^ = 0.900), PROTEIN (R^2^ = 0.888), and SNF (R^2^ = 0.883) are notable for a compact 18-band sensor operating in the 410–940 nm range. Very low-density errors (MAE ≈ 0.00174, RMSE ≈ 0.00246) further show that the system offers not only high correlation but also error magnitudes compatible with physical measurement precision.

The relative ordering of the five algorithms under LOO (XGB and GB best, RF a close third, ADA clearly weaker, KNN weakest) is consistent across all six targets and has a straightforward interpretation. Gradient boosting (GB and XGB) exploits the fact that milk-composition targets are locally smooth non-linear functions of the 18 channels; each boosting round corrects the residual of the previous ensemble and, therefore, captures fine-grained interactions (e.g., between IR_3 and VIS_4 in the FATNESS target). Random Forest offers comparable accuracy but averages many deep trees and, therefore, smooths away some of the sharpest interactions. AdaBoost, which up-weights difficult samples through reweighting rather than residual fitting, is more sensitive to label noise in the 25–75% adulteration levels and plateaus earlier. KNN, a distance-based learner with no explicit feature weighting, cannot down-weight the uninformative UV channels in the presence of strong IR/VIS signal and, therefore, trails the tree ensembles by 15–20 percentage points in R^2^. XGBoost’s additional L2 regularization and column subsampling further explain its slight edge over plain GB on targets with wide dynamic range (FATNESS, ADDED WATER, FREEZING).

The comparative literature summary in Table 11 indicates that previous AS7265x-based studies reported high accuracy with limited band selection. For example, one study reported R^2^ = 0.933 for protein and R^2^ = 0.997 for fat using six wavelengths [14], but with a limited sample size (n = 100). Another study used 18 channels in the 410–940 nm range but did not include XAI-based band analysis, and its validation strategy was less strict than LOO [8]. In this context, the present work provides a more conservative but more reliable performance evaluation via both a larger sample size (n = 190) and LOO validation.

To contextualize the accuracy of the proposed AS7265x system against established measurement modalities, four method classes can be compared. (i) Wet-chemistry reference methods such as Gerber for fat (ISO 2446) [17] and Kjeldahl for protein (ISO 8968-1) [18] achieve the highest absolute accuracy but require trained personnel, hazardous reagents, and 30–90 min per sample—they are a laboratory ground-truth, not a field instrument. (ii) Benchtop FTIR analyzers (e.g., MilkoScan/FOSS) deliver R^2^ ≥ 0.98 for fat and R^2^ ≥ 0.94 for protein but cost 30–60 k€ and are not portable. (iii) Portable ultrasonic analyzers (e.g., Milkana Superior Plus, used here as the reference instrument) cost approximately 1.5–3 k€ and deliver ±0.1% accuracy for fat, ±0.2% for protein and SNF, and ±0.0005 g/cm^3^ for density within their certified range, but they degrade markedly outside it (e.g., ±5% for added water) and require a 15 mL aspirated sample per measurement. (iv) On-farm NIR sensors (960–1690 nm) reach R^2^ ≈ 0.98 for fat and R^2^ ≈ 0.94 for protein but cost 5–10 k€ per installation. The proposed AS7265x system, at a hardware cost of approximately 40–60 USD per unit, delivers LOO R^2^ = 0.922 for fat, 0.888 for protein, 0.883 for SNF, 0.877 for density, 0.900 for freezing point, and 0.892 for added water—that is, within 5–8 percentage points of the R^2^ reported for benchtop FTIR and on-farm NIR systems while simultaneously estimating six parameters on a single reading with no moving parts, no reagents, and a measurement time of a few seconds. The practical positioning is therefore that of a screening and adulteration-alert instrument deployable at every step from collection tank to factory intake, where current commercial solutions are prohibited by cost or throughput.

In an AS7263-based study by Wang et al., R^2^ = 0.867 for protein and R^2^ = 0.971 for fat were reported [15]; however, limited band count and lack of XAI reduce spectral interpretability. In SciO-based systems, high values such as R^2^ = 0.969 for fat and R^2^ = 0.917 for protein were reported, but those studies used small sample sizes (n = 45) and market label declarations as references [12], which may introduce calibration uncertainty.

Higher-accuracy values such as R^2^ ≈ 0.98 for fat and R^2^ ≈ 0.94 for protein have also been reported in broader-band NIR (960–1690 nm) or FTIR systems. However, those are typically laboratory-grade, high-cost solutions requiring calibration against reference instruments (e.g., MilkoScan/FOSS) [13]. In contrast, this study achieves comparable explanatory levels with a portable, low-cost multispectral sensor operating in the 410–940 nm range, offering an important cost-performance advantage.

The repeated dominance of IR_3 (approximately 860 nm) across all target variables provides a strategic design implication. The near-infrared region around 860 nm is a critical spectral window for both water absorption and Mie scattering linked to fat globules. The key role of IR_3 in ADDED WATER, FREEZING, and DENSITY aligns with water-driven physical mechanisms, while its high contribution in FATNESS and PROTEIN indicates that this wavelength captures multi-component interactions. Although band selection has been reported in the literature, systematic reporting of band importance together with physical interpretation remains limited [8,14,15]. In this work, XAI analyses explicitly reveal the band-physical mechanism relationship. Moreover, while many studies target a single quality parameter, this study models six quality parameters simultaneously on the same sensor platform. Compared with prior work, this multi-parameter approach provides a clear methodological and practical advantage. Overall, the proposed 18-band AS7265x-based method offers a strong and holistic alternative through (i) larger sample size with LOO validation, (ii) XAI supported wavelength/feature selection, (iii) physically interpretable band-contribution analysis, and (iv) single-platform estimation of multiple quality parameters.

### 3.8. Five-Fold Cross-Validation Results and Comparison with LOO

To complement the sample-level LOO evaluation, we repeated the full pipeline (with fold-internal IsolationForest outlier filtering and fold-internal preprocessing) using stratified 5-fold KFold (shuffle = True, random_state = 42). The per-target best-model summary is reported in Table 12. The two validation strategies produce consistent conclusions: in every target, the best-performing model under LOO remains the best or within 0.01 R^2^ of the best under 5-fold, and the rank order of feature sets (RAW > FUSION ≈ DERIVED) is preserved. The 5-fold R^2^ values are typically within ±0.02 of the LOO values, with absolute MAPE differences below 2 percentage points for fat, protein, SNF, density, and freezing point. The agreement between the two protocols indicates that our performance estimates are not an artifact of a particular validation choice, and that the model does not benefit from the larger training fold that LOO provides—i.e., the learning curve is close to saturation at N ≈ 150.

## 4. Conclusions

In this study, multi-parameter estimation of milk composition and adulteration indicators was performed using a low-cost AS7265x-based multispectral electronic tongue system. In experimental analyses conducted on a total of 190 augmented milk samples, fat, protein, SNF, density, freezing point, and added-water parameters were modeled on the same sensor platform. Different feature sets (RAW, DERIVED, and FUSION) and multiple machine-learning algorithms were comparatively evaluated, and model performance was tested using both five-fold cross-validation and the LOO strategy.

The findings showed that a narrow-band and cost-effective optical system can reliably predict not only basic components but also adulteration-sensitive parameters. Across all six target variables, the RAW 18-channel feature set consistently provided the best or most balanced LOO performance (e.g., fat R^2^ = 0.922, freezing point R^2^ = 0.900, protein R^2^ = 0.888, SNF R^2^ = 0.883, density R^2^ = 0.877, and added water R^2^ = 0.892 with XGB or GB). The DERIVED feature set alone was systematically weaker, and combining RAW with DERIVED (FUSION) did not yield an additional improvement over RAW, indicating that the physically informative signal already resides in the 18 narrow spectral channels and that derivative/statistical post-processing introduces additional variance rather than new information in this sample size. Permutation-based importance analysis within the XAI framework enabled separation of critical spectral bands for each quality parameter and produced data-driven insights for sensor design. In this regard, the study not only presented a performance-oriented prediction model but also developed a band-based optimization perspective for low-cost sensor design.

However, to improve field-level generalizability of the system, it is important to expand dataset diversity in terms of geography, season, and producer. Model robustness can be tested more comprehensively with broader datasets including different heat-treatment types, feeding conditions, and quality classes. Based on XAI results, band-reduction studies can investigate whether similar performance can be maintained with fewer spectral channels. This approach may provide critical advantages in hardware cost and energy consumption.

In future work, model architecture can be extended with more advanced versions of current regression algorithms and alternative ensemble strategies, together with deeper parameter-optimization studies. In addition, development of lightweight edge-compatible models is important for integrating the system into an IoT-based continuous quality-monitoring chain from farm to factory. Accordingly, development of explainable and reliable AI-based intelligent milk quality monitoring platforms is a natural continuation of this study. As a promising direction suggested during the peer-review process, Bayesian Regularized Artificial Neural Networks (BRANN) can complement the tree-ensemble family used here. BRANN applies a Bayesian prior directly to the network weights and can therefore deliver well-calibrated predictions on small chemometric datasets where deterministic deep networks would over-fit. For a 190-sample regression problem with 18 narrow-band inputs, BRANN is expected to be competitive with gradient boosting while additionally offering per-prediction uncertainty estimates—a useful property for adulteration-alert applications, where one wants the model to flag not only the predicted water-addition percentage but also its confidence in that estimate. A systematic comparison between tree-ensemble predictors and BRANN (including a combined stacking architecture in which BRANN provides a prior-informed meta-predictor over the tree ensembles) is left for future work.

## Figures and Tables

**Table 1:** Sample distribution across adulteration levels (N = 190).

Dilution Ratio (%)	Sample
0	52
2	8
3	8
4	8
5	8
10	8
20	8
25	20
30	15
40	15
50	15
60	15
75	15

**Table 2:** Basic statistics of target variables (N = 190).

Parameter	Variable	Min	Max	Mean	Std. Dev.
Fat (g/100 mL)	FATNESS	0.16	3.40	1.85	0.95
Protein (g/100 mL)	PROTEIN	0.79	3.15	2.11	0.77
Solids-Not-Fat (g/100 mL)	SNF	1.86	8.34	5.52	2.09
Density (g/mL)	DENSITY	1.0048	1.0287	1.0180	0.0075
Freezing Point (°C)	FREEZING	−0.549	−0.053	−0.341	0.159
Added Water Amount (%)	ADDED WATER	1.22	90.30	38.59	28.62

**Table 3:** AS7265x spectral channels and central wavelengths.

Variable	Wavelength (nm)
asUV_0	410
asUV_1	435
asUV_2	460
asUV_3	485
asUV_4	510
asUV_5	535
asVIS_0	560
asVIS_1	585
asVIS_2	610
asVIS_3	645
asVIS_4	680
asVIS_5	705
asIR_0	730
asIR_1	760
asIR_2	810
asIR_3	860
asIR_4	900
asIR_5	940

**Table 4:** Machine Learning Parameters.

Component	Parameters
5-Fold	n_splits = 5, shuffle = True, random_state = 42
Isolation Forest	n_estimators = 1000, contamination ∈ {0.00, 0.03, 0.05, 0.07, 0.10}, random_state = n_jobs = −1
Random Forest	n_estimators = 1200, random_state = 42, n_jobs = −1
Gradient Boosting	random_state = 42
AdaBoost	n_estimators = 400, learning_rate = 0.05, random_state = 42
KNN	n_neighbors = 7, weights = distance
XGBoost	objective = reg:squarederror, n_estimators = 1800, reg_lambda = 1.0, random_state = 42, n_jobs = −1; additionally, max_depth and learning_rate were selected on a small grid
Permutation Importance	n_repeats = 30, scoring = neg_root_mean_squared_error, random_state = 42

**Table 5:** Comparison of performance metrics for added water prediction.

FEATURE SET	MODEL	R^2^	MAPE (%)	MAE	RMSE
RAW	XGB	0.892	57.200	6.143	8.783
RAW	GB	0.891	58.758	6.316	8.824
RAW	RF	0.855	69.159	7.539	10.158
FUSION	GB	0.853	65.800	7.500	10.105
FUSION	XGB	0.849	69.634	7.544	10.246
RAW	ADA	0.835	90.442	9.357	10.858
DERIVED	GB	0.814	88.430	8.915	11.797
DERIVED	XGB	0.813	84.562	8.825	11.841
FUSION	ADA	0.804	90.255	9.761	11.682
FUSION	RF	0.791	88.403	9.064	12.046
DERIVED	RF	0.777	97.583	9.776	12.921
DERIVED	ADA	0.749	119.782	11.704	13.715
RAW	KNN	0.715	111.443	11.132	14.255
FUSION	KNN	0.706	118.002	11.585	14.293
DERIVED	KNN	0.670	129.379	12.443	15.729

**Table 6:** Comparison of performance metrics for density prediction.

Feature Set	Model	R^2^	MAPE (%)	MAE	RMSE
RAW	GB	0.877	0.171	0.002	0.002
RAW	XGB	0.868	0.177	0.002	0.003
FUSION	GB	0.853	0.198	0.002	0.003
FUSION	XGB	0.836	0.207	0.002	0.003
RAW	RF	0.834	0.206	0.002	0.003
DERIVED	GB	0.809	0.234	0.002	0.003
RAW	ADA	0.807	0.259	0.003	0.003
DERIVED	XGB	0.787	0.239	0.002	0.003
FUSION	RF	0.780	0.245	0.002	0.003
FUSION	ADA	0.773	0.274	0.003	0.003
DERIVED	RF	0.756	0.266	0.003	0.004
DERIVED	ADA	0.722	0.325	0.003	0.004
RAW	KNN	0.680	0.302	0.003	0.004
FUSION	KNN	0.669	0.318	0.003	0.004
DERIVED	KNN	0.656	0.332	0.003	0.004

**Table 7:** Comparison of performance metrics for fat prediction.

FEATURE SET	MODEL	R^2^	MAPE (%)	MAE	RMSE
RAW	XGB	0.922	12.378	0.189	0.261
RAW	GB	0.907	13.277	0.207	0.285
FUSION	XGB	0.895	15.698	0.213	0.302
FUSION	GB	0.894	15.921	0.220	0.305
RAW	RF	0.891	16.718	0.231	0.309
RAW	ADA	0.859	22.975	0.297	0.350
FUSION	ADA	0.858	22.295	0.296	0.353
FUSION	RF	0.857	20.244	0.270	0.353
DERIVED	XGB	0.857	18.369	0.249	0.353
DERIVED	GB	0.848	18.994	0.258	0.363
DERIVED	RF	0.813	21.814	0.295	0.403
RAW	KNN	0.773	23.971	0.339	0.445
DERIVED	ADA	0.772	27.560	0.369	0.445
FUSION	KNN	0.752	26.050	0.368	0.466
DERIVED	KNN	0.709	28.583	0.401	0.503

**Table 8:** Comparison of performance metrics for freezing-point prediction.

FEATURE SET	MODEL	R^2^	MAPE (%)	MAE	RMSE
RAW	XGB	0.900	11.520	0.033	0.047
RAW	GB	0.892	11.878	0.035	0.049
FUSION	GB	0.857	15.198	0.041	0.055
RAW	RF	0.856	15.145	0.042	0.056
FUSION	XGB	0.849	15.331	0.042	0.057
RAW	ADA	0.840	18.738	0.051	0.059
FUSION	ADA	0.795	21.670	0.056	0.066
FUSION	RF	0.794	18.778	0.050	0.067
DERIVED	GB	0.793	17.773	0.049	0.067
DERIVED	XGB	0.793	18.616	0.050	0.067
DERIVED	RF	0.746	22.180	0.057	0.074
RAW	KNN	0.715	22.492	0.062	0.079
DERIVED	ADA	0.709	26.484	0.068	0.079
FUSION	KNN	0.707	23.495	0.064	0.079
DERIVED	KNN	0.648	25.887	0.069	0.087

**Table 9:** Comparison of performance metrics for protein prediction.

FEATURE SET	MODEL	R^2^	MAPE (%)	MAE	RMSE
RAW	GB	0.888	8.546	0.170	0.240
RAW	XGB	0.885	8.345	0.167	0.243
FUSION	GB	0.861	9.970	0.197	0.265
RAW	RF	0.848	10.513	0.207	0.280
FUSION	XGB	0.848	10.392	0.203	0.277
RAW	ADA	0.825	13.167	0.257	0.301
DERIVED	XGB	0.796	12.673	0.244	0.326
DERIVED	GB	0.795	12.654	0.245	0.327
FUSION	ADA	0.783	14.455	0.273	0.330
FUSION	RF	0.779	12.894	0.251	0.333
DERIVED	RF	0.752	14.429	0.274	0.360
DERIVED	ADA	0.718	17.316	0.328	0.383
RAW	KNN	0.699	15.816	0.307	0.394
FUSION	KNN	0.687	16.483	0.320	0.396
DERIVED	KNN	0.631	18.414	0.348	0.438

**Table 10:** Comparison of performance metrics for SNF prediction.

FEATURE SET	MODEL	R^2^	MAPE (%)	MAE	RMSE
RAW	GB	0.883	9.494	0.482	0.668
RAW	XGB	0.878	9.323	0.472	0.682
FUSION	GB	0.855	10.866	0.545	0.738
FUSION	XGB	0.845	11.190	0.561	0.765
RAW	RF	0.841	11.635	0.583	0.780
RAW	ADA	0.820	14.297	0.713	0.829
DERIVED	GB	0.787	13.410	0.663	0.896
DERIVED	XGB	0.785	13.587	0.670	0.901
FUSION	RF	0.777	13.779	0.691	0.916
FUSION	ADA	0.775	15.653	0.757	0.920
DERIVED	RF	0.733	15.978	0.768	1.004
DERIVED	ADA	0.697	18.954	0.920	1.068
RAW	KNN	0.691	17.399	0.858	1.086
FUSION	KNN	0.687	17.569	0.875	1.086
DERIVED	KNN	0.628	19.096	0.935	1.185

**Table 11:** Comparison with related works.

Ref.	Sensor	Wavelength (nm)	XAI	Number of Samples	Performance Metric
[14]	AS7265x	610/680/730/760/810/860	Wavelength selection	100	Protein R^2^ = 0.933 Fat R^2^ = 0.997
[8]	AS7265x	410–940	Yok	600+	Fat MAPE = 0.14 Protein MAPE = 0.07
[15]	AS7263	610/680/730/760/810/860	Yok	60	Protein R^2^ = 0.8677 Fat R^2^ = 0.9713
[12]	SCiO	740–1070 1350–2550	Yok	45	Fat R^2^ = 0.969 Protein R^2^ = 0.917 Carbohydrate R^2^ = 0.883
[13]	On-farm NIR sensor	960–1690	Spectral band selection	1165	Fat R^2^ = 0.98 Protein R^2^ = 0.94 Lactose R^2^ = 0.84
Proposed Method	AS7265x	410–940	Wavelength and feature selection	190	Added Water R^2^ = 0.892 Density R^2^ = 0.877 Fat R^2^ = 0.922 Freezing Point R^2^ = 0.900 Protein R^2^ = 0.888 SNF R^2^ = 0.883

**Table 12:** Five-fold cross-validation results.

FEATURE SET	TARGET	MODEL	R^2^	MAPE (%)	MAE	RMSE
RAW	FATNESS	GB	0.915	14.909	0.198	0.276
FUSION	FATNESS	GB	0.899	17.019	0.211	0.300
RAW	FREEZING	XGB	0.872	14.447	0.039	0.057
RAW	PROTEIN	XGB	0.868	9.977	0.194	0.278
RAW	ADDED WATER	GB	0.863	75.507	7.835	10.551
DERIVED	FATNESS	XGB	0.851	21.796	0.257	0.365
RAW	SNF	GB	0.848	12.276	0.585	0.812
DERIVED	ADDED WATER	GB	0.844	92.153	8.514	11.290
RAW	DENSITY	XGB	0.840	0.201	0.002	0.003
DERIVED	SNF	GB	0.839	13.225	0.618	0.837
FUSION	ADDED WATER	XGB	0.838	96.876	8.316	11.490
DERIVED	FREEZING	GB	0.838	19.878	0.047	0.064
DERIVED	PROTEIN	GB	0.837	12.402	0.226	0.308
FUSION	FREEZING	XGB	0.834	17.776	0.045	0.065
FUSION	PROTEIN	XGB	0.832	11.612	0.219	0.312
FUSION	SNF	XGB	0.826	12.867	0.619	0.869
DERIVED	DENSITY	GB	0.818	0.228	0.002	0.003
FUSION	DENSITY	XGB	0.807	0.228	0.002	0.003

## Data Availability

The dataset can be shared with the corresponding author upon request with justification.
